# Is “disease management” the answer to our problems? No! Population health management and (disease) prevention require “management of overall well-being”

**DOI:** 10.1186/s12913-016-1765-z

**Published:** 2016-09-21

**Authors:** Jane Murray Cramm, Anna Petra Nieboer

**Affiliations:** Institute of Health Policy & Management (iBMG), Erasmus University, Rotterdam, The Netherlands

**Keywords:** Chronic disease, Disease management, Quality of life, Well-being, Self-management, Health behavior

## Abstract

**Background:**

Disease management programs based on the chronic care model have achieved successful and long-term improvement in the quality of chronic care delivery and patients’ health behaviors and physical quality of life. However, such programs have not been able to maintain or improve broader self-management abilities or social well-being, which decline over time in chronically ill patients. Disease management efforts, population health management initiatives and innovative primary care solutions are still mainly focused on clinical and functional outcomes and health behaviors (e.g., smoking cessation, exercise, and diet) failing to address individuals’ overall quality of life and well-being. Individuals’ ability to achieve well-being can be assessed with great specificity through the application of social production function (SPF) theory. This theory asserts that people produce their own well-being by trying to optimize the achievement of instrumental goals (stimulation, comfort, status, behavioral confirmation, affection) that provide the means to achieve the larger, universal goals of physical and social well-being.

**Discussion:**

A shift in focus from the management of physical function, disease limitations, and lifestyle behaviors alone to an approach that fosters self-management abilities such as self-efficacy and resource investment as well as overall quality of life, is urgently needed. Disease management interventions should be aimed at adequately addressing all difficulties chronically ill patients face in life, such as the effects of pain and fatigue on the ability to maintain a job and social life and to participate in activities promoting physical and social well-being. Patients’ ability to maintain engagement in stimulating work and social activities with the people who are important to them may be even more important than aspects of disease self-management such as blood pressure or glycemic control. Interventions should aim to make chronically ill patients capable of managing their own well-being and adequately addressing their needs in a broader sense.

**Summary:**

So, is disease management the answer to our problems in the time of aging populations and increased prevalence of unhealthy lifestyles, chronic illnesses, and comorbidity? No! Effective (disease) prevention, disease management, patient-centered care, and high-quality chronic care and/or population health management calls for *management of overall well-being*.

## Background

The complexity of chronic disease profiles, population health management, disease prevention, promotion of healthier lifestyles all demand a patient-centered system of care delivery characterized by *long-term coordination* among *diverse* health professionals. Such a patient-centered disease management approach is expected to equip patients with the information and skills necessary to act as capable self-managers and thereby improving patient outcomes [1]. Chronic illnesses, however, continue to be underdiagnosed and undertreated, and approaches to their care rarely combine primary measures (i.e., prevention of disease onset) with preventive secondary measures (i.e., treatment of patients with known risk factors or in the initial stages of disease) [2]. In the United States, increasingly common efforts to transform primary care centers into patient-centered medical facilities have achieved only limited improvements in care quality, implying that further refinement of such interventions is needed [3]. Changes are needed in population health management strategies, the way chronic care is delivered, how diseases are prevented as well as how we assess their outcomes. Research to date suggests strongly that such shifts require multicomponent interventions, such as disease management programs based on the chronic care model [1, 4, 5]. Such programs target patient populations in which significant positive effects of interventions to improve self-care have been demonstrated [4–8].

The chronic care model was developed to guide the redesign and quality improvement of chronic care delivery. Its multidimensional framework outlines the transformation of care from an acute, reactive approach to a planned, population-based strategy rooted in productive interaction between informed, activated patients and proactive health care teams [6–8]. The model defines six interrelated components of the quality of chronic care delivery:

1. self-management support (empowering patients to self-manage care through planning, goal setting, and problem solving, e.g., with education and guided development of skills, healthy lifestyle, and self-efficacy)
2. delivery system design (defining health care team members’ roles and delivering evidence-based care that patients understand)
3. decision support (making care decisions with patients using evidence-based guidelines and specialists’ expertise)
4. clinical information systems (providing timely reminders for patients and health professionals, planning and coordinating care, monitoring health care team performance)
5. health care organization (promoting effective strategies at all levels to comprehensively change the care system, developing agreements to coordinate care and address quality issues, providing incentives to improve care quality), and
6. community linkages (developing partnerships with community organizations to support interventions that complement health services, advocating for policy changes that improve patient care) [4–6].

Based on these components, primary care practices implementing the chronic care model deliver care in a manner that activates and involves patients, improves care coordination and evidence-based decision making, and enables monitoring of the effectiveness of care for individual patients, with the overall aim of improving the quality of care. By focusing on clinical and functional outcomes and health behaviors (e.g., smoking cessation, exercise, and diet) [9–11], however, this model largely fails to address patients’ overall quality of life and well-being [12]. Interventions are needed to change chronically ill patients’ behavior and engage them in activities that promote improvement in physical (e.g., functioning, pain management, general health) and mental (e.g., vitality, social functioning, psychological health) quality of life [13, 14]. Care providers should thus prioritize improvement of chronically ill patients’ quality of life while treating or managing illness and impairment [9]. Thus, interventions aiming to maintain or improve chronically ill patients’ well-being by preventing disease onset, promoting healthier lifestyles and social engagement, and aiding the development of self-management abilities are an important complement to population health management [15–18].

### Management of overall well-being

Individuals’ ability to achieve well-being can be assessed with great specificity through the application of social production function (SPF) theory. SPF theory, developed by Lindenberg [19], is based on the concept that people make diverse efforts to improve their living conditions, with the overall aim of achieving physical and social well-being. It assumes that as a society, we try to protect well-being by providing care and support to those who depend on it, for example because of functional limitations. The best organisation of such care depends on the manner in which it contributes to well-being. Thus, the determinants of well-being and the best approaches to improving it must be established.

**Fig. 1:**
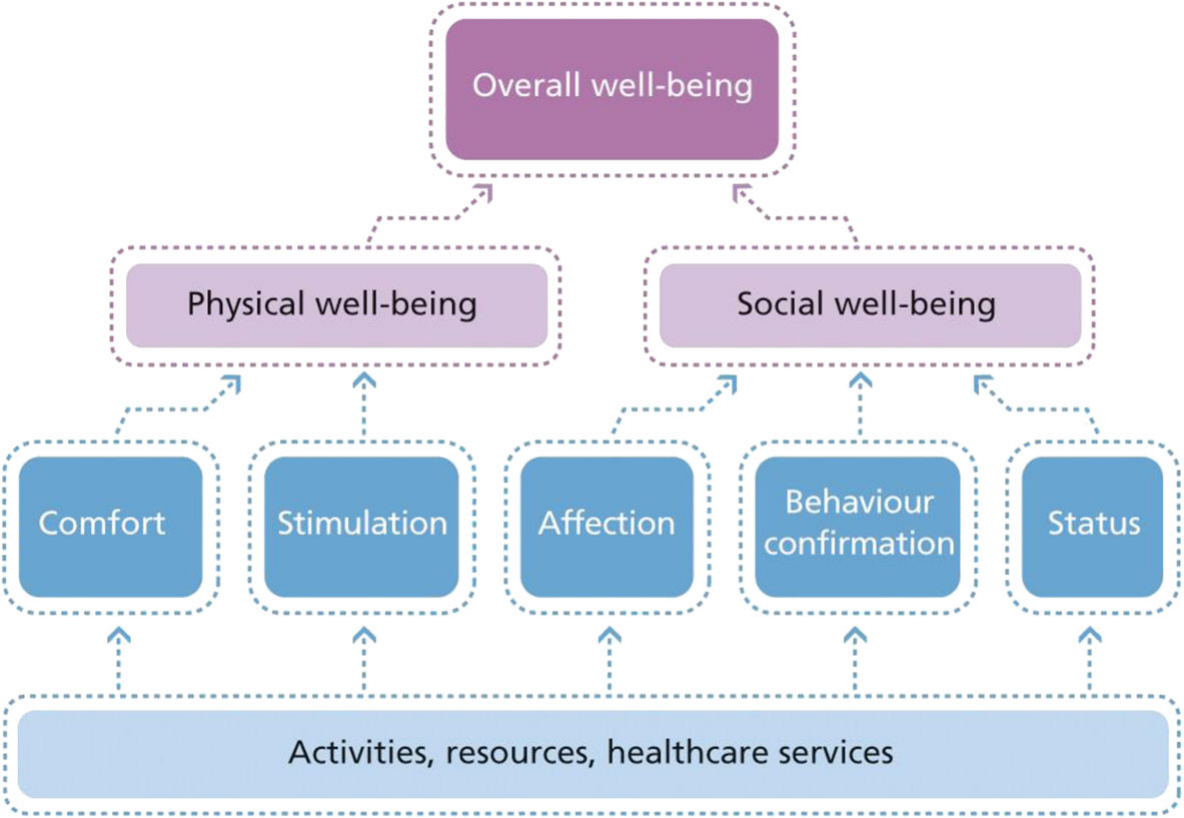
The hierarchy of well-being, according to social production function theory. From: [46] Cramm JM, Nieboer AP: Social cohesion and belonging predict the well-being of community-dwelling older people. BMC Geriatrics 2015. 15:30. DOI:10.1186/s12877-015-0027-y

Well-being is a broad concept with physical and social dimensions. Physical well-being is a construct involving optimal *comfort* and adequate physical and mental *stimulation*. The state of comfort, which has somatic and emotional components, comprises the presence of a safe, pleasant environment and the absence of physiological needs (i.e. pain, hunger, and thirst). Social well-being can be achieved by obtaining *status* (social ranking, e.g. based on occupation, lifestyle, or talents), *behavioural confirmation* (living according to relevant others’ or one’s own norms), and *affection* (friendship, intimacy, and emotional support, e.g. from a partner, children, or loved ones) [20, 21]. Physical and social well-being are achieved on the way to the ultimate goal of overall subjective well-being (optimal quality of life or mental well-being) [21]. By recognising the hierarchy of well-being goals, we can better understand the impacts of chronic illnesses and the functional limitations that come with it, and thereby determine the types of care and support that individuals require. This theory thus asserts that people produce their own well-being by trying to optimise the achievement of instrumental goals (stimulation, comfort, status, behavioural confirmation, affection) that provide the means to achieve the larger, universal goals of physical and social well-being (Fig. 1) [9]. Nieboer [21] developed a valid, reliable instrument to assess all five instrumental goals (stimulation, comfort, status, behavioural confirmation, and affection) to achieve well-being based on the SPF theory. Those aiming to measure and improve well-being based on the SPF theory can use this instrument.

Illness and functional limitations affect well-being in diverse ways. A person’s resources (e.g. physical condition, social relationships, and income) have been shown to affect well-being in times of illness and of health. For example, social contacts and the myriad forms of support and confirmation received from a partner have important buffering functions [22].

The ability to participate in activities that are important to an individual, an essential component of physical and social well-being, is fostered by social relationships and other resources. Not all activities are equal; withdrawal from multifunctional activities (i.e. those that contribute to multiple goals in the well-being hierarchy) can have a substantial impact on the ability to maintain or achieve well-being. For example, physical exercise can contribute to physical well-being, but engaging in exercise with others (e.g. in a sports club) can also enhance social well-being. Functional limitations leading to withdrawal from important activities thus have major impacts on well-being, unless an individual has the opportunity to substitute for losses. For people with functional limitations, the provision of care and support that facilitate continued engagement in important activities can reduce or avoid loss of well-being [9].

## Discussion

Early studies showed that disease management programs and other interventions based on the chronic care model improved patients’ health behaviors, thereby preventing decline [10, 23–25]. We (Cramm and Nieboer) [23] have documented the challenges within such programs of achieving real gains in terms of patients’ overall well-being. Beyond patients’ self-management of their chronic conditions, interventions have not been able to effectively motivate patients to become proactive participants in care delivery or to self-manage well-being in a broader sense. These challenges reflect the failure of disease management interventions to adequately address the difficulties facing chronically ill patients, such as the effects of pain and fatigue on the ability to maintain a job and social life and to participate in activities promoting physical and social well-being. Patients’ ability to maintain engagement in stimulating work and social activities with the people who are important to them may be even more important than aspects of disease self-management such as blood pressure or glycemic control. Results from a meta-analytic review for example showed a 50 % increased likelihood of survival for people with stronger social relationships [26]. In the larger context of well-being, self-management abilities are known to deteriorate as a consequence of dealing with chronic illness [27], and disease management programs based on the chronic care model have not been able to achieve a shift in this pattern [10, 23]. The implementation of such a program in the Netherlands led to improved physical quality of life, but a decline in mental quality of life [23, 24]. The development of truly patient-centered systems will thus require prioritization of the overall quality of life and well-being of chronically ill patients.

As broader self-management abilities are critical predictors of well-being and mental quality of life [16, 23, 28–30], a shift in focus to include not only traditionally addressed health- and disease-specific aspects (e.g., smoking, physical activity, blood pressure monitoring), but also abilities such as investment behavior (e.g., pursuing interests, keeping busy, maintaining contact with loved ones) and self-efficacy (e.g., belief in one’s ability to achieve goals and express care for others), is urgently needed [31]. Patients living with chronic illness experience not only functional and clinical impacts, but also compromised quality of life due to factors such as anxiety and fear about the impacts of the illness on themselves, their loved ones, and their financial status [32, 33]. Health care professionals’ attention to such worries and concerns and investment in strengthening patients’ ability to cope with them are thus of great importance. Chronically ill patients value personal contact with these professionals highly, as it gives them the opportunity to talk about their concerns; such contact cannot be replaced by e-consultation, the development of patient portals for online information exchange, or similar interventions, which convey the notion that patients must “do everything themselves.” In the implementation of disease management programs, these alternative forms of contact have been found to be insufficient to stop declines in self-management abilities and mental quality of life as consequences of living with chronic illness [23].

Despite achieving improvement in the quality of chronic care delivery, disease management programs in the Netherlands and United States have not been able to successfully address self-management support or mental quality of life issues [23, 34–36]. Approaches to self-management support are the least frequently implemented and most challenging elements of the chronic care model [37], and Elissen and colleagues [38] demonstrated that such support for patients with chronic illness is far from adequate in most European countries. A better understanding of how patients and health care professionals can be encouraged to promote prevention and engage in productive interaction is thus clearly required to improve chronically ill patients’ physical as well as social well-being. We expect that the implementation of interventions to strengthen patients’ investment behavior and self-efficacy would be beneficial, but such an approach requires a different perspective on care delivery. Although there are some examples out there aimed at improving one or two of the instrumental goals to improve well-being, there are no examples of programs aiming to improve all five instrumental goals to improve well-being. Furthermore, evidence about these initiatives is still largely lacking. Using the SPF-IL scale [9] assessing the five instrumental goals to achieve well-being may help to create empirical evidence. Health care professionals must strive to understand each patient’s context [39] and be responsive to his or her needs, values, and preferences, rather than perceiving him or her solely as the object of disease [40]. Berwick [41] conceptualized effective care delivery as that involving health care professionals in the role of “guests” in patients’ lives, rather than “hosts” in the health care system. Such approaches have been shown to improve patient outcomes [42], but they require professionals to interact effectively with patients, gaining and using knowledge of them as complete, unique people [43]. Most health care professionals have not received training in the communication skills and psychological counseling techniques needed to achieve such interaction [44, 45].

## Conclusions

Disease management programs based on the chronic care model have achieved successful and long-term improvement in the quality of chronic care delivery [34–36] and patients’ health behaviors [10, 23] and physical quality of life [23, 24]. However, such programs have not been able to maintain or improve broader self-management abilities or mental quality of life, which decline over time in chronically ill patients [23, 27, 38]. A shift in focus from the management of physical function, disease limitations, and lifestyle behaviors alone to an approach that fosters self-management abilities such as self-efficacy and resource investment as well as overall quality of life, is urgently needed. Health care professionals must also realize the importance of personal contact with patients to discuss concerns about dealing with chronic illness. However, the implementation of interventions that meet patients’ needs while enhancing their self-management abilities and making them proactive participants in care delivery poses a challenge. The extension of chronically ill patients’ self-care abilities for as long as possible is becoming increasingly important, as better self-management can prevent disease worsening and maintain patients’ independence and physical and mental quality of life. In addition to directly benefitting individual patients, these achievements would reduce demands on overburdened health care systems and improve overall population health. Disease management programs have not yet discovered how to effectively help chronically ill patients become informed, activated self-managers. Patient-centered interventions should aim to make these patients capable of managing their own health and quality of life, thereby improving overall well-being and adequately addressing their needs. So, is disease management the answer to our problems in the time of aging populations and increased prevalence of unhealthy lifestyles, chronic illnesses, and comorbidity? No! Effective (disease) prevention, disease management, patient-centered care, and high-quality chronic care and/or population health management calls for *management of overall well-being*.

## Abbreviation

SPF: Social production function
